# Experimental Demonstration of Compact Polymer Mass Transfer Device Manufactured by Additive Manufacturing with Hydrogel Integration to Bio-Mimic the Liver Functions

**DOI:** 10.3390/bioengineering10040416

**Published:** 2023-03-26

**Authors:** Ganesan Narendran, Avdhoot Walunj, A. Mohan Kumar, Praveen Jeyachandran, Nasser S. Awwad, Hala A. Ibrahium, M. R. Gorji, D. Arumuga Perumal

**Affiliations:** 1 Micro and Nanofluidics Laboratory, Department of Mechanical Engineering, National Institute of Technology Karnataka, Suruthkal, Mangaluru 575025, India; 2 Department of Mechanical Engineering, Mahatma Phule Krishi Vidyapeeth, Rahuri 413722, Maharashtra, India; 3 Department of Clinical Microbiology, All India Institute of Medical Sciences, Mangalagiri 522503, Andhra Pradesh, India; 4 Department of Mechanical Engineering, National Institute of Technology Karnataka, Suruthkal, Mangaluru 575025, India; 5 Department of Chemistry, College of Science, King Khalid University, P.O. Box 9004, Abha 61413, Saudi Arabia; aawwad@kku.edu.sa; 6 Department of Biology, College of Science, King Khalid University, P.O. Box 9004, Abha 61413, Saudi Arabia; habrahem@kku.edu.sa; 7 Faculty of Medicine and Health Sciences, Ghent University, 9000 Ghent, Belgium

**Keywords:** liver function, biomimicry, additive manufacturing, hydrogel, selective laser sintering

## Abstract

In this paper, we designed and demonstrated a stimuli-responsive hydrogel that mimics the mass diffusion function of the liver. We have controlled the release mechanism using temperature and pH variations. Additive manufacturing technology was used to fabricate the device with nylon (PA-12), using selective laser sintering (SLS). The device has two compartment sections: the lower section handles the thermal management, and feeds temperature-regulated water into the mass transfer section of the upper compartment. The upper chamber has a two-layered serpentine concentric tube; the inner tube carries the temperature-regulated water to the hydrogel using the given pores. Here, the hydrogel is present in order to facilitate the release of the loaded methylene blue (MB) into the fluid. By adjusting the fluid’s pH, flow rate, and temperature, the deswelling properties of the hydrogel were examined. The weight of the hydrogel was maximum at 10 mL/min and decreased by 25.29% to 10.12 g for the flow rate of 50 mL/min. The cumulative MB release at 30 °C increased to 47% for the lower flow rate of 10 mL/min, and the cumulative release at 40 °C climbed to 55%, which is 44.7% more than at 30 °C. The MB release rates considerably increased when the pH dropped from 12 to 8, showing that the lower pH had a major impact on the release of MB from the hydrogel. Only 19% of the MB was released at pH 12 after 50 min, and after that, the release rate remained nearly constant. At higher fluid temperatures, the hydrogels lost approximately 80% of their water in just 20 min, compared to a loss of 50% of their water at room temperature. The outcomes of this study may contribute to further developments in artificial organ design.

## 1. Introduction

The liver is a complex organ with several essential roles in the synthesis, detoxification, and regulation of blood components in the human body, its malfunction poses a threat to our life [1]. Liver failure can emerge without any prior liver disease; typically, it can be due to intoxication or extreme decompensation [2,3]. Some of the major symptoms of liver failure are icterus, hepatic encephalopathy, and an impairment of coagulation, and these acute conditions could lead to multiorgan failure [4]. If the liver fails to regenerate, the only definitive treatment is orthotopic liver transplantation [5]. Since donor organs are rare, especially for patients who are not identified for high-priority transplantation, many may not survive to when an appropriate donor organ is available. Additionally, the side effects of contraindications further prevent liver transplantation in many cases [6]. For these situations, extracorporeal liver assistance devices have been developed, in order to either temporarily support the damaged organ until it can regenerate or bridge the patient to transplantation [7]. In severe liver dysfunction, toxins might build up in the blood and lead to hepatic encephalopathy, potentially leading to multi-organ failure [8]. This can be addressed by artificial devices similar to dialysis [9] and blood oxygenator [10] systems. These devices work by recapitulating liver functions within a synthetic liver that is capable of adsorption and filtering as an artificial liver support device [11]. There are several extracorporeal toxin-removal techniques, such as hemodialysis, continuous venovenous hemofiltration (CVVH), hemoperfusion, and molecular adsorbent recirculating systems (MARS) [12,13].

The most common treatment used to remove water-soluble toxins from the blood is hemodialysis [14]. In this process, blood is diffused through a semipermeable membrane with dialysates flowing in the opposite direction from the extracorporeal circuit’s blood flow. The clearance of a toxin during hemodialysis is influenced by the membrane type and surface area, as well as the blood and dialysate flow rates. The primary limitations of the technique are hemodynamic stability and the risk of redistribution of the toxins [15]. In charcoal haemoperfusion, the patient’s plasma is separated and passed through activated charcoal filters that can adsorb a range of water-soluble toxins in the low and intermediate molecular weight range, which are responsible for liver failure [16]. Initial issues of these devices, such as biocompatibility, thrombocyte loss, and clotting issues, were resolved by preventing direct contact between the plasma and charcoal particles [17]. In the case of plasmapheresis, a plasma filter is used to separate the blood’s cellular components from the plasma. The plasma is substituted with albumin and frozen plasma with other plasma constituents [18]. This process eliminates several toxins that are present in the plasma, however, this approach carries the danger of infections and requires a large quantity of plasma. More complex detoxification mechanisms were developed to manage many toxins, which included the removal of lipophilic, albumin-bound toxins [19]. The two key principles that emerge from this are albumin dialysis and fractionated plasma separation.

The MARS relies on the recycling of albumin solution using active charcoal and an anion exchanger [20]. Blood from the patient is pumped through the hollow fiber capillaries of a dialysis filter. Albumin solution passes through the membrane counter-directionally, allowing albumin-bound toxins in the blood to become trapped in the MARS device, however, albumin cannot get through the membrane [21]. Toxins are removed by the filter after passing through the adsorber and filter cartridges, and albumin is then regenerated and becomes ready to receive additional toxins after another membrane passage. Additionally, CVVH dialyzes the albumin from the circuit itself, reducing a load of water-soluble toxins [22]. A similar concept is used in single-pass albumin dialysis (SPAD); blood from the patient also flows across a high-flow dialysis membrane [23]. Toxins from the plasma are taken up by an albumin solution as it flows anticlockwise along the opposite side of the membrane. In SPAD, however, the albumin solution is thrown away after passing through the membrane just once, without being regenerated. This permits CVVH to utilize the same dialysis filter. In an in vitro investigation, the effectiveness of eliminating both albumin-bound and water-soluble toxins by MARS, SPAD, and CVVH was compared [24,25,26]. For all three systems, there was no discernible difference in the elimination of water-soluble pollutants. Both MARS and SPAD eliminated albumin-bound toxins to a similar degree, demonstrating a substantially higher efficiency than CVVH. Even though all of the aforementioned techniques led to better biochemical and clinical conditions and the removal of toxins, they failed to demonstrate an advantage for the patients’ mortality. To overcome this in recent times, liver tissue engineering (LTE) has been extensively studied, in order to create liver models that closely resemble the functions of an in vivo liver as possible.

There are two primary applications for LTE: first, bioengineered livers can be utilized as in vitro models for evaluating xenobiotics, toxicological research, and patient-specific disease models. This is achieved by using hydrogels [27,28], which are one of the most promising alternatives for supportive biomaterials [29,30,31], and have been widely used in tissue engineering and regenerative medicine [32]. Table 1 presents a summary of the different hydrogels used in biomedical applications. A hydrogel is a network of hydrophilic polymer chains that can be either natural or synthetic, and has a degree of elasticity that is comparable to that of natural tissues. A detailed evaluation of the characteristics of various hydrogels is required to imitate the liver extracellular matrix (ECM), which is critical for liver cell engraftment, long-term survival, and function [33]. Hydrogels are also known as stimuli-responsive hydrogels, or smart polymers, because they alter their structure and chemical characteristics in reaction to external environmental factors, such as temperature, pH, solvent composition, electric field, and ionic strength [34,35,36]. Especially, temperature-sensitive hydrogels are of significance for drug delivery because of their solubility characteristics, which change from sol to gel in response to external temperature [37,38]. The temperature has impressive control over the interaction of water molecules’ hydrophobic and hydrophilic polymeric chains. As a result, at a critical temperature, hydrogels go through a volume phase transition, specifically, a lower critical solution temperature (LCST) and an upper critical solution temperature (UCST). At higher temperatures, the LCST hydrogel exhibits a hydrophilic to hydrophobic transition, but the UCST hydrogels show the opposite shift [39]. Due to its capacity to load medication at lower temperatures, the LCST hydrogel is used more often than UCST. The most commonly used LCST hydrogel is poly (*N*,*N*-diethyl acrylamide) (PDEA), which has an LCST of 31 °C [40]. Ramanan et al. [41] investigated a polymer-based, temperature-sensitive hydrogel, poly (N-isopropyl acrylamide) (PNIPAAm). They demonstrated that bovine serum albumin (BSA) can be released in response to temperature changes. Sun et al. [42] developed a hydrogel film by blending chitosan and temperature-sensitive PNIPAAm and polyethylene glycol (PEG) for temperature and pH characterization. The blended hydrogel, which had an LCST of 32 °C, was shown to develop more porosity regions at a higher temperature of 37 °C. The release rate was greatly influenced by the medium’s pH and salt concentrations at 37 °C, which affect how effectively the hydrogel amino groups interface with the NS-2 sulfonic groups [43]. The controlled release of adriamycin was studied by Qiao et al. [44] using thermosensitive micelles made of poly (L-alanine) and PNIPAAm. It was reported that in the adriamycin kinetic experiments, 25% of the adriamycin was released within 50 h at a temperature of 25 °C. As a result, at 37 °C, nearly 70% of doxorubicin (DOX) was released. 

From the aforementioned literature, it can be concluded that several studies have been reported to recapitulate the function of the liver; in particular, several extracorporeal toxin-removal devices work primarily on the membrane and hollow fibers. Apart from that, in a greater perspective, the functions of the liver are not just confined only to adsorption and filtration; functions such as the synthesis and regulation of compounds are also equally important to partially fulfill as a synthetic organ. Similarly to this, several hydrogel-related works have been presented to mimic the controlled drug release and functional LTE. However, very rarely are these hydrogels placed in a state-of-the-art device, in order to examine the behavior at dynamic work conditions, which is required for the development of artificial organs. In this present study, we demonstrate a proof-of-concept of an additively manufactured device, in which a hydrogel is integrated that functions with temperature and pH stimuli. In particular, the device independently regulates the temperature (LCST), for the release of methylene blue (MB) from the hydrogel at varying flow rates.

## 2. Biomimicry Design and Manufacturing

**Figure 1:**
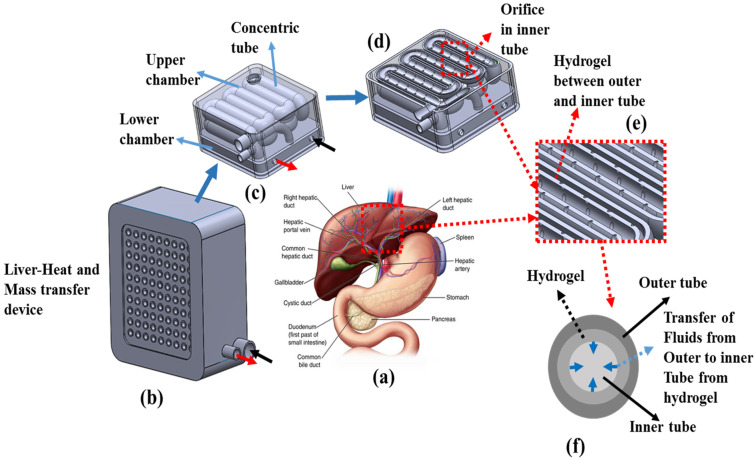
Schematics of the device design evolution. (**a**) Sketch details of the human liver with blood sources and glands, (**b**) 3D model of the actual additive manufactured device, with a fluid inlet and outlet, comprising concentric tubes, (**c**) the internal sections of the device, with a concentric tube in the upper section and lower section for the inlet and outlet of the device, (**d**) sliced portion of the concentric tube showing the orifice in the inner tube, (**e**) detailed section, highlighting the hydrogel between the outer and inner tube, and (**f**) the cross-section of the concentric tube where the mass transfer occurs, from the outer to inner tube.

In this study, the compact liver mass transfer device was developed using additive manufacturing technology (AM), performed by adding materials layer upon layer [50], as shown in Figure 1. Figure 1a depicts the details of the liver with its sections. The liver is located in the upper right-hand portion of the abdominal cavity, beneath the diaphragm, and on top of the stomach, right kidney, and intestines. It has two large sections, called the right and left lobes [51]. The gallbladder sits under the liver, along with the pancreas and intestine. These organs work together to digest, absorb and process food. The liver regulates most of the chemical levels in the blood and excretes a product called bile [52]. Furthermore, all the blood leaving the stomach and intestine passes through the liver. The liver processes this blood, and breaks down molecules, creates nutrients, and also metabolizes drugs into non-toxic forms that are are easily used in the body. The liver has been identified to have more than 500 vital functions. Two of the very well known functions include the following; (a) regulating blood amino acid levels, which form the building blocks of proteins, and (b) the liver additionally detoxifies the blood and maintains healthy blood sugar levels, providing nutrients to the parts of the body [53]. Two distinct sources supply blood to the liver: oxygenated blood flows from the hepatic artery, and nutrient-rich blood flows from the hepatic portal vein. The hepatic portal vein is a blood vessel that carries blood from the gastrointestinal tract and spleen to the liver, in order to pick up nutrients and toxins from the stomach and intestine for further processing [54]. The blood received from the intestine contains carbohydrates, fats, vitamins, and other nutrients dissolved in it. The functional unit of the liver is the lobule. The oxygen-rich blood from the hepatic artery mixes with nutrient rich blood in the lobule cells before leaving in the central vein. For our biomimicry design, based on the liver, we have taken two different sources/fluids (s_1_, s_2_) and two functions (f_1_, f_2_) for heat and mass transfer applications. In the first function (f_1_), the fluid (s_1_) temperature is regulated using forced convection and in the second function (f_2_), fluid (s_2_) is defused into the temperature-regulated fluid (s_1_) and exits the device.

Here, we introduce the proof-of-concept of using AM to develop a biomimicry microsystem that recapitulates the functional intricacy of the human liver for mass transfer application, as shown in Figure 1b. Especially, we developed a liver heat and mass transfer (LHMT) model by using selective laser sintering (SLS)-based AM technology to construct a compartmentalized section, in order to reproduce two functions, consisting of an upper and lower chamber completely made of Nylon (PA-12) shown in Figure 1c. For the formation of the LHMT model, two compartment sections have to work harmoniously. In particular, the lower section performs heat transfer and then the fluid is fed into the mass transfer section of the upper compartment. The upper chamber has the serpentine concentric tube; here, the diffusion of s_2_ to s_1_ takes place in the counter flow direction. Figure 1d depicts the sectional view of the concentric serpentine tube, with Figure 1e showing the orifice in the inner tube. Hydrogel is present between the outer and inner tubes of the serpentine concentric tube, and it facilitates the transfer of fluid (s_2_) from the outer tube to the inner tube fluid (s_1_) through the orifice, as shown in Figure 1f.

**Figure 2:**
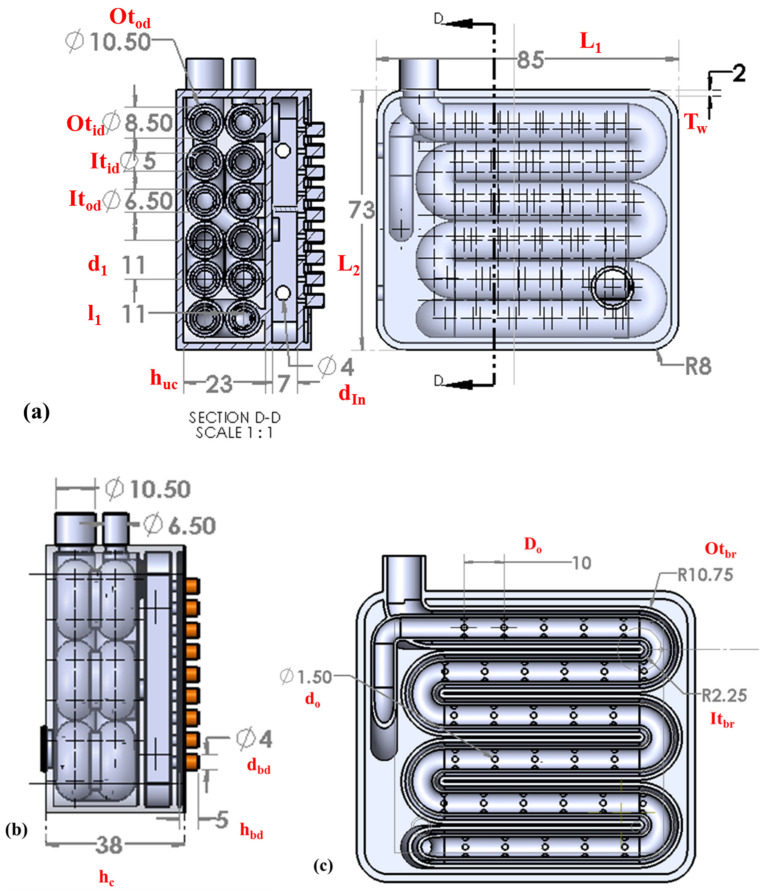
3D schematic representation of the additively manufactured device’s geometry, with its dimensions. (**a**) Top view with cut section, (**b**) side view, and (**c**) sectional view.

Figure 2 shows the schematic representation of the AM device’s geometry, with its dimensions. As shown in Figure 1, the device comprises two chambers. The hot fluid flow occurs in the bottom chamber. The fluid for the inlet of the serpentine tube is taken from the bottom chamber. The total size of the chamber is 73 mm × 85 mm, and the height of the chamber is 38 mm, as shown in Figure 2a. The length of the serpentine pattern is 49 mm and the wall thickness of the chamber is 2 mm. The concentric tubes’ inner and outer diameters are 10.50 mm and 6.50 mm, respectively, as shown in Figure 2b. The inner tube has an orifice with a 1.50 mm diameter, which is used in the diffusion process, as shown in Figure 2c. Table 2 presents the design parameters of the liver heat and mass transfer device, with its dimensions.

**Figure 3:**
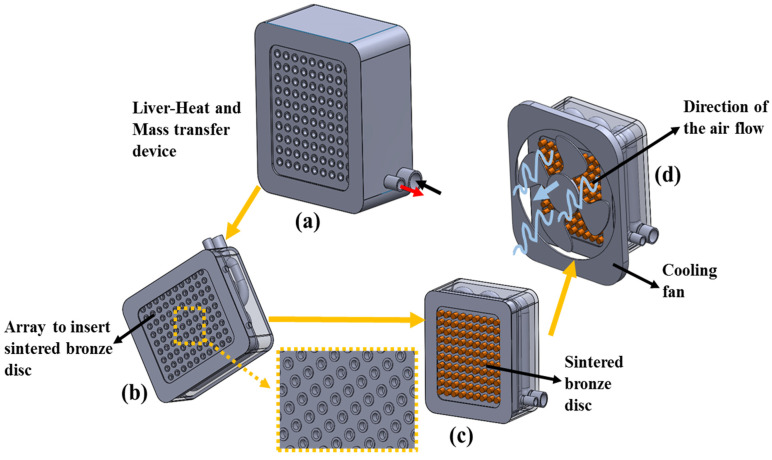
Schematics of the temperature regulation system. (**a**) Bottom side of the liver heat and mass transfer device, showing the lower chamber, (**b**) the lower chamber of the device, showing an array used to insert a sintered bronze disc, (**c**) the lower chamber of the device with the sintered bronze disc, and (**d**) the device attached to the cooling fan and showing the direction of the airflow.

Figure 3 presents the temperature regulation system. Figure 3a shows the liver heat and mass transfer device from the bottom side of the lower chamber. For the formation of the heat transfer section, the fluid–air is interfaced at the bottom surface of the device, and seeded with an array of a sintered bronze disc, as shown in Figure 3b,c. Figure 3d depicts the attachment of the cooling fan, with the flow direction. This arrangement is very much essential to perform function (f_1_), i.e., to regulate the s_1_ temperature to the required hydrogel response temperature. This temperature regulation arrangement facilitates the diffusion of source s_2_ from the hydrogel to s_1_.

**Figure 4:**
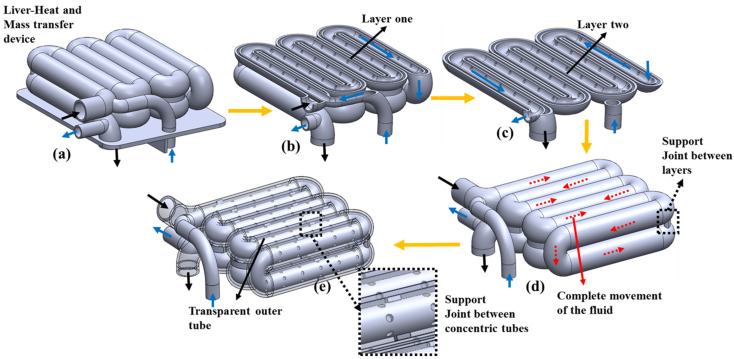
Schematics of the concentric tubes of LHMT device. (**a**) Upper chamber of the concentric tube with a chamber partition wall and with details of the lower chamber’s concentric tube inlet and outlet partition wall. (**b**) Cut section of layer one of the concentric tube, (**c**) cut section of layer two of the concentric tube, (**d**) details of the complete movement of the fluid in the concentric tube, with the highlighted details of the solid support joint between layer one and layer two, and (**e**) transparent view of the outer wall of the concentric tube, showing the inner wall, with the details of the solid support joint between the concentric tubes.

Figure 4 presents the schematics of the concentric tube of the LHMT device. Figure 4a presents the upper chamber concentric tube and the chamber’s partition wall, with details of the lower chamber’s partition wall. First of all, the chamber’s partition wall serves as a separation wall between the temperature regulation and hydrogel diffusion process. The inlet and outlet of the concentric tube are present in the lower chamber and separated by another partition wall to avoid mixing. The cut section of layer one of the concentric tube is shown in Figure 4b. Both s_1_ and s_2_ meet in layer one and pass through the concentric serpentine tube, as highlighted by the arrows’ directions. The cut section of layer two of the concentric tube is shown in Figure 4c. the outlet of the outer concentric tube is delivered to the lower chamber and the outlet of the inner tube is taken outside of the device. Figure 4d shows the complete movement of the fluid in the two layers of the concentric tube, with the highlighted details of the solid support joint between the layers. Figure 4e shows the transparent view of the concentric tube, showing the inner concentric tube, with the details of the solid joint between the concentric tubes for support.

## 3. Materials, Methods, and Manufacturing

### 3.1. LHMT Device Fabrication

**Figure 5:**
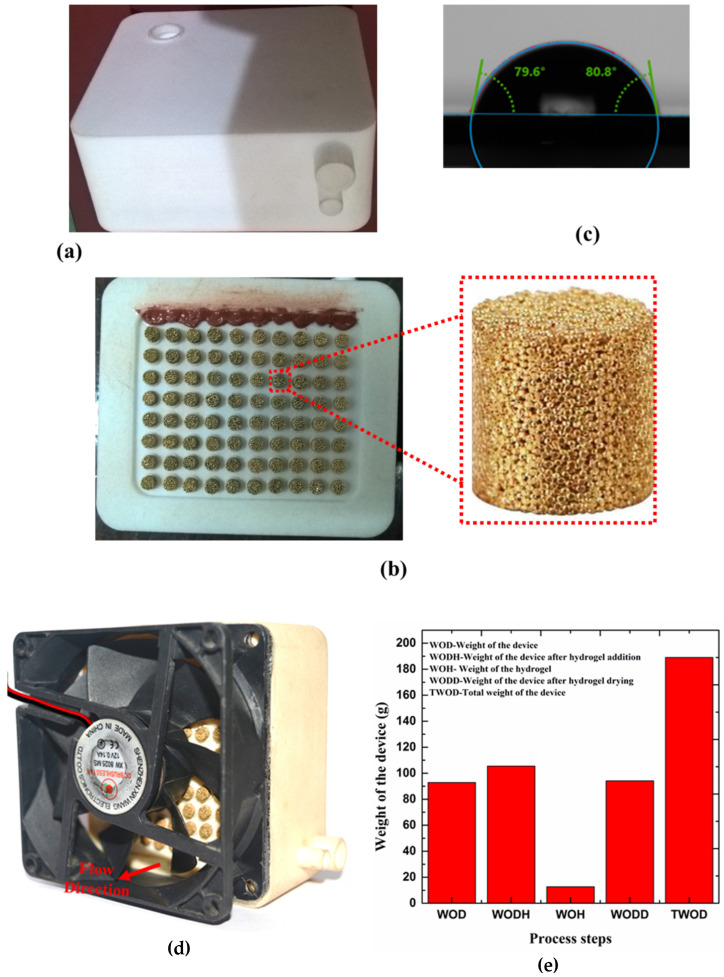
Actual picture of the additively manufactured liver heat and mass transfer. (**a**) Additively manufactured device, front view, (**b**) Additively manufactured device, bottom view, with a sintered bronze disc attached to the array; highlighted section shows the actual bronze sintered disc used in the study, (**c**) contact angle image of the additively manufactured nylon substrate with water, (**d**) complete photograph of the device, showing the airflow direction, and (**e**) weight of the device after different process steps in the experiment.

The selective sintering process (SLS) is a versatile AM process that creates 3D parts by focusing a laser beam onto a thin powder layer in 2D patterns. The powder is melted and fused into a solid part by absorbing electromagnetic radiation from the emitted laser. This process is repeated layer-by-layer on the fused powder to generate 3D structures. A major advantage of SLS technology is in constructing an overhanging structure by using unfused power as a support structure for subsequent layers. SLS has better resolution because it uses a focused laser to print sub-millitmeter features. The Nylon 12 (PA 650, Advanced laser materials, TX, USA) powder used in this study was sourced from local SLS printing using HP MJF 4200 (Akaar Technologies, Banglore, India), as shown in Figure 5. Figure 5a shows the front view of the actual experimental device. Figure 5b shows the additively manufactured device, bottom view, with a sintered bronze disc attached to the array; the highlighted section shows the actual bronze sintered disc used in the study. The 3D-printing machine has an external dimension size of 2210 × 1200 × 1448 mm. A CO_2_ laser was used with 100 W power, at a job process resolution of 600 dpi. The part modeling was done using Solidworks and converted to STeroLithography (STL) format for 3D printing. The plastic is a fine white powder of approximately 30 µm in diameter, with a melting temperature of 175–190 °C.

The contact angle of the 3D printed substrate is in the range of 79°, as shown in Figure 5c. Figure 5d shows the complete photograph of the device, showing the airflow direction. Figure 5e presents the weight of the device after different process steps in the experiment. The nylon powder is loaded into the feeder and electronic code instructions are programmed. Then, a laser source delivers a high-powered beam toward a reflective mirror. Later, a galvanometer motor system directs the source to the powder source. From here, the geometry is sintered from a single layer by developing a nylon melt pool. Then, the feeder is moved up to repeat the next layer. This process is repeated layer-by-layer until the build is complete. The minimum wall thickness of 0.8–1.2 mm, maximum hole details of 1.5 mm, a layer thickness of 0.08–0.01 mm, standard accuracy of ±0.3%, grainy matt surface finish, and excess powder are removed in post-processing. Then, the finished LHMT device is subjected to a jet of air and fine glass beads to remove the excess nylon powder from the device’s surface, as well as from inside the device. Finally, a burst of compressed air is used to remove any blasted media left behind in the device.

### 3.2. Hydrogel Synthesis and Integration

From Tokyo Chemical Industry co., Ltd. (TCI), Tokyo, Japan, we purchased *N*,*N*-diethyl acrylamide (DEA, 98%). A 99% concentration of *N*,*N*’-methylbisacrylamide (MBA) was bought from Loba Chemie in Mumbai, India. Merck Specialities Pvt. Ltd., Mumbai, India, supplied the ammonium peroxodisulfate (APS, 98%). Tetramethyl ethylenediamine (TEMED, 99%) was bought from HI Media Laboratories Pvt. Ltd. In Mumbai, India. Using free radical polymerization, the Poly (*N*,*N*-diethyl acrylamide) (PDEA) hydrogel was produced. In a brief, TEMED (50 mL) was introduced, dropwise, to a beaker containing known amounts of the monomer DEA (2.179–15.2 mmol), the cross-linker MBA (129.7 mol), the initiator APS (87.67 mol), and milli-Q water (5 mL). For 4 h, the polymerization reaction was carried out at 28 °C, the standard room temperature. At the end of the synthesis, 5 mL of the solution was injected into the serpentine tube (in between the walls). The Inlet and outlet were covered with a paraffin film and allowed to settle for 24 hrs. Then, the entire device was lowered into distilled water for 48 hrs to remove the residual chemicals. After that, the gadget was vacuum-dried, in order to get rid of the water inside. The device was weighed when it had finished drying, and the weight added by the hydrogel was recorded. The pump and sensors were then connected to the device’s inlet and outlet, which are attached to the test rig. For the initial study, normal, distilled water of a known volume, at room temperature, was circulated in the serpentine tube to study the Increased weight of the hydrogel due to reswelling. The reswelling Is given by the ratio; SR = (Wt – W_d_)/Ws, where Wt is the weight of the gel at the specified time t, Ws is the weight of water In the swollen gel, and W_d_ is the dry weight of the gel. In the following experiment, a constant temperature bath was used to raise the fluid temperature between the ranges of 30 °C and 38 °C. The remperature response of the hydrogel was investigated using pH and methylene blue (MB) indicators. The inlet and outlet of the concentric tube were connected to the pump with a known concentration of MB solution (5 mg/L), with the pump operating for 12 hrs (at room temperature). Then, distilled water was circulated to remove the excess MB solution (note that the washed water MB concentration is accounted for volume calculation). To investigate the controlled release of the hydrogel, the temperature bath was set to a temperature ranging from 30 °C (below LCST) to 37 °C (above LCST). Measurements of the outlet were taken at regular intervals with a UV-vis spectrometer. The release can be presented as a cumulative release, with the ratio CR% = Mt/Mq × 100, as a function of time, where Mq is the estimated amount of dye loaded in the hydrogels and Mt is the quantity of dye released from the hydrogels at time t. The same procedure was followed for the pH measurement using saline solution. At different temperatures, the fluid was circulated. The outlet was studied in terms of the pH and MB concentrations. The results are shown as cumulative release as a function of time, where CR = M_T_/Mq × 100 and MT represents the amount of dye released from the hydrogels at a given temperature and the estimated amount of dye loaded in the hydrogels, respectively. The error estimation was performed multiple times.

### 3.3. Experimental Flow Line

**Figure 6:**
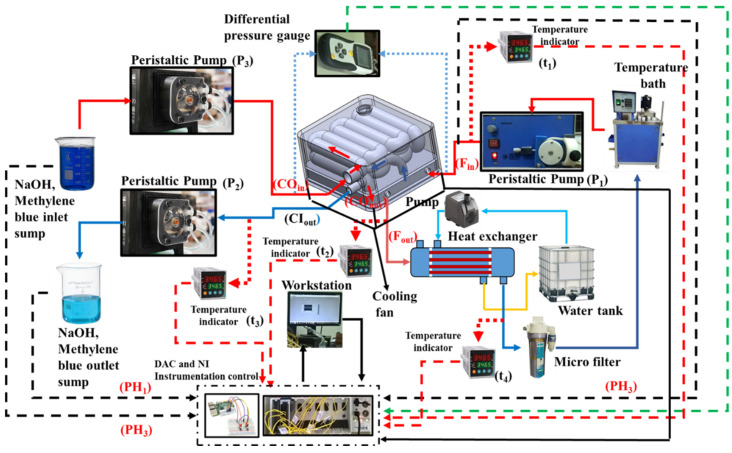
Schematics of the experimental flow line with respective components and sensor used.

Figure 6 depicts the components used for the experiment with the flow line. The flow line includes a peristaltic pump, workstation, thermocouples, differential pressure transducers, data acquisition system, breadboard, raspberry pi controller, control valves, heat exchanger, temperature bath, cooling fan, temperature controller, and temperature indicator. The experimental flowline of the devices can be summarized as follows: the inlet (F_in_) is delivered to the lower chamber of the device using a peristaltic pump (P_1_), connected to a temperature bath. The distilled water (Millipore) is circulated from a temperature bath with a temperature range from 30 °C to 42 °C. The fluid inlet was monitored using a temperature indicator (t_1_). The flow of fluid into the upper chamber starts from the inner tube of the concentric pipe inlet (CI_in_). The negative pressure of the inner tube of the serpentine tube was delivered at the outlet (CI_out_) by pump (P_2_) and, the pressure was measured using a differential pressure gauge. The outlet of the pump (P_2_) is directed to the sump; here, the outlet fluid pH was monitored by a pH sensor (Extech 6012B) (pH_1_), and temperature is monitored using a temperature indicator (Digiqual systems, Chennai, India) (t_3_). The hydrogel saline concentration in the outer tube is periodically maintained by pump (P_3_), with an inlet (CO_in_) in the upper chamber. The outlet (CO_out_) of the outer tube is fed into the lower chamber. The fluid from the device exits at the outlet (F_out_), and the temperature was monitored using the temperature indicator (t_2_). Finally, the device output connects with the heat exchanger and the microfilter, containing the pH modulator, and sends it back to the temperature bath. The bottom of the device is equipped with a cooling fan to regulate the inlet temperature following the hydrogel response. The national instruments (NI) data acquisition system is connected to all of the temperature sensors and pH sensors. K-type thermocouples were used for the experiment with NI-9213, in order to acquire the voltage signal, and the pH voltage signal was acquired using NI-9215. All the pumps are connected with the hardware interface module raspberry pi and interfaced with LabVIEW, installed in the workstation. The upper and lower values of temperature and pH are given in the LabVIEW panel, and were used to set the operating conditions. To start the experiment, the LabVIEW front panel is initialized. All the pumps and sensors are connected so that all the devices are online in the front panel. For P_3_, the sump is filled with 0.001 M of NaOH (pH = 10). For the first case, the (t_1_) temperature is set at 34 °C and the temperature bath is set at 37 °C. Then, the pump is initialized, and hot fluid reaches the lower chamber. If the temperature of t_1_ >34 °C, the cooling fan activates and lowers the temperature to 34 °C. This initiates the pump P_2_ with negative pressure, and at the same time, pump P_3_ is also initiated, with the condition that the flow rate of P_3_ << P_2_. The outlet of the P_2_ was constantly monitored for pH and samples were monitored with a UV-vis spectrometer. This same procedure was followed for different t_1_ temperatures of 32 °C to 37 °C; similarly, the flow rate of pump P_2_ varies from 10–50 mL/min and the pH of P_3_ is varied from 8 to 12.

## 4. Results and Discussion

**Figure 7:**
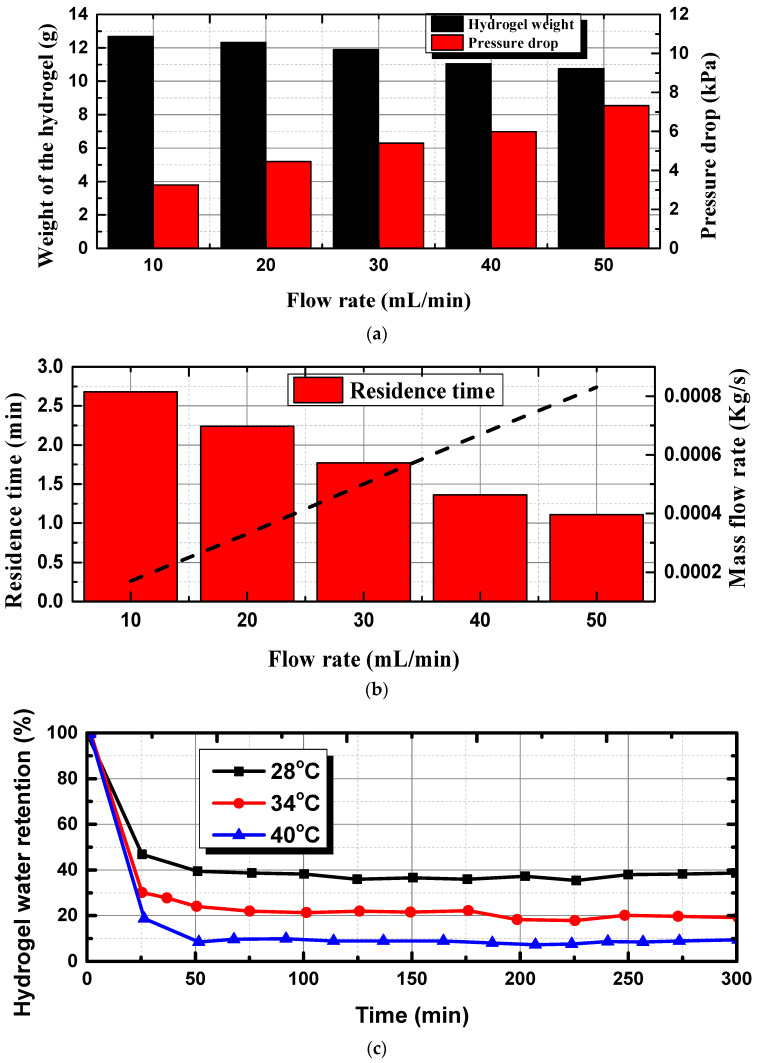

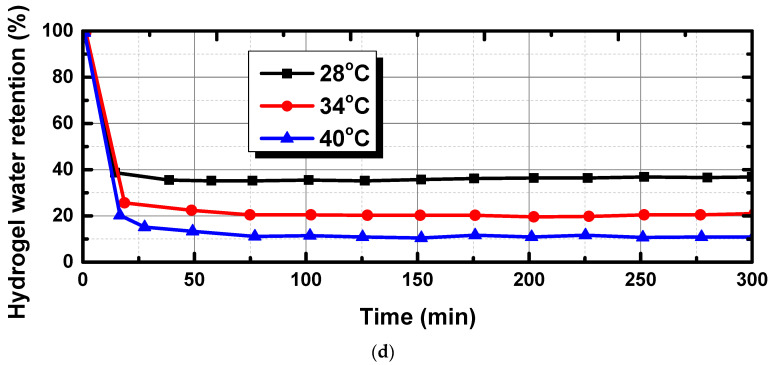
Weight of the hydrogel and residence time as a function of flow rate, and hydrogel water retention over time. (**a**) Secondary axis, showing the increment in pressure drop as a function of flow rate, (**b**) the corresponding mass flow rate is given in the secondary axis, (**c**) hydrogel water retention as a function of time for different fluid temperatures, at a constant flow rate of 10 mL/min, and (**d**) hydrogel water retention as a function of time for different fluid temperatures, at a constant flow rate of 50 mL/min.

Figure 7 depicts the pressure drop and residence time as a function of flow rate (mL/min) and hydrogel water retention over time. Figure 7a depicts the weight of the hydrogel (g) as a function of flow rate, and the increase in pressure drop with the increase in flow rate. The weight of the hydrogel reached its maximum at 10 mL/min and decreased by 25.29%, to 10.12 g, for the flow rate of 50 mL/min. On other hand, the increase in flow rate to 50 mL/min resulted in the maximum pressure drop of 7.21 kPa. To address this in detail, the residence time, as a function of flow rate, is presented in Figure 7b. These results indicate that a lower flow rate promotes the development of a higher residence time of 2.62 min, and at 50 mL/min, this time is just 1 min. From critical observation, it can be found that the effect of weight gain of the hydrogel due to swelling at an increased flow rate is lesser than the increased pressure drop. At a lower flow rate, the hydrogel’s swelling over time is reflected in the increase in the weight of the hydrogel. We observed this behavior due to the pre-constructed walls that alter the swelling characteristics, due to which the hydrogel ultimately did not reach the final volume. A similar effect on hydrogel selling in confined conditions was observed by Louf et al. [55]. Figure 7c presents the hydrogel water retention percentage as a function of time, at three different temperatures for a constant flow rate of 10 mL/min. The synthetic hydrogel deswelling kinetics were observed at temperatures between room temperature (28 °C) and 50 °C. The results show that hydrogels tested at higher fluid temperatures (40 °C) were quicker to de-swell and lost more water over time when compared to hydrogels tested at lower fluid temperatures (28 °C). At higher fluid temperatures, hydrogels lost approximately 80% of their water in just 20 min, compared to 50% at room temperature. It was observed that after 50 min, the hydrogel water retention capacity was unaltered for all three temperature conditions. When 40 °C water interacts with hydrogels, the hot fluid passes from the inner concentric tube to the hydrogel outermost region. This would cause the hydrophilic interactions between the hydrophobic groups to intensify, which eventually causes the outermost surface to rapidly contract and produce a thick, skin-like layer. The hydrogel water molecules cannot diffuse out after this skin has developed, which causes a delayed response time. This explains why the hydrogel water retention was nearly constant 50 min after the experiment. With the hydrogel’s dense layer acting as a resistor to the liquid flow during the deswelling process, the deswelling ratio was low. In the case of a higher flow rate, the decrease in the hydrogel water retention was steeper, and achieved a constant retention percentage within 30 min, as shown in Figure 7d. As discussed in Figure 7a, the results can be attributed to the increased pressure drop in the serpentine tubes, which may have thickened the hydrogel layer instantaneously.

### Temperature-Dependent Hydrogel Study

**Figure 8:**
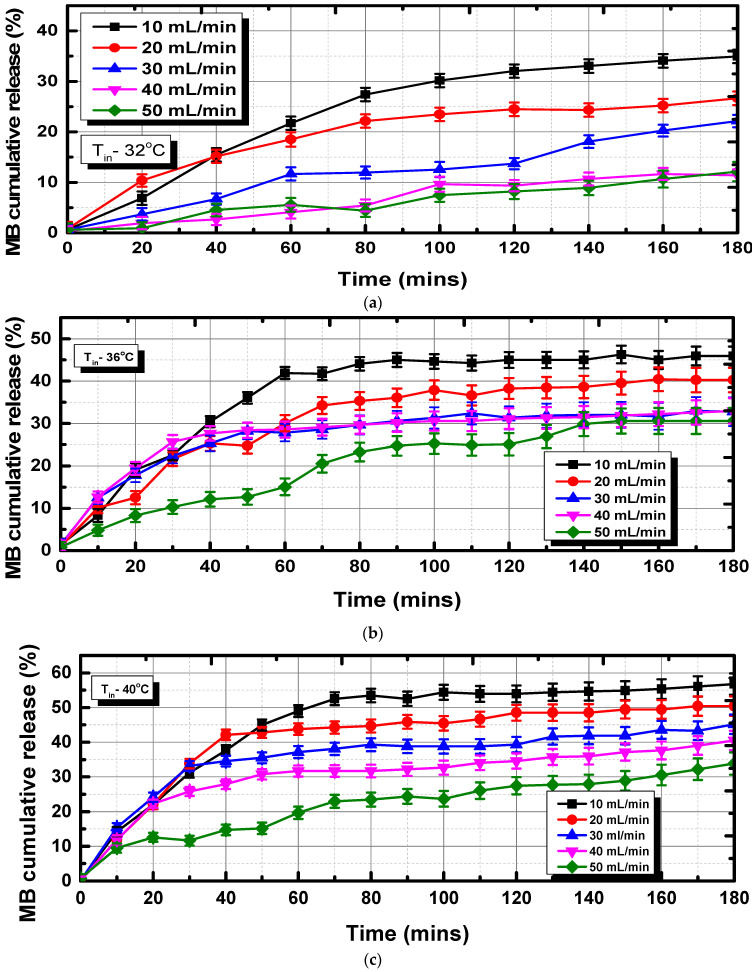
MB cumulative release as a function of time at different temperatures: (**a**) 32 °C, (**b**) 36 °C, and (**c**) 40 °C.

The amount of MB released from the outlet was periodically measured using UV/Vis spectroscopy at 680 nm. The results are presented in terms of cumulative release (C_R_; C_R_ = M_t_/M_α_ × 100) as a function of time. MB release from the polymer matrix is tightly correlated with several variables, including drug affinity to polymer chains, drug release, drug solubility in water, and polymer matrix swelling behavior. This study uses the water-soluble thiazine dye methylene blue (MB) as a model medication. The hydrogel samples were used to measure the cumulative release of MB (5 ppm). The final equilibrium weights of the dry sample (12.68 g) and of the 100 mL of the 5 ppm MB solution were calculated to be 14.05 + 0.06 g. The difference between the concentration of MB solution at the beginning and end of the experiment revealed an intriguing behavior of the hydrogels, showing that they could accumulate MB molecules. From Figure 8, it is clear that the amount of MB released by hydrogels varies depending on temperature. According to Figure 8a, the cumulative MB release at 32 °C is 37% for the lower flow rate of 10 mL/min, but in the case of 50 mL/min, the MB release drastically reduced to 10%. For the case of 36 °C, the MB cumulative release reached up to 44% at a lower flow rate of 10 mL/min, as shown in Figure 8b. The cumulative release at 40 °C climbed to 55%, which is 44.7% more than at 30 °C, as shown in Figure 8c. The temperature increased the MB cumulative release percentage. Additionally, MB release in every sample fell as the flow rate increased. Maximum MB release from the hydrogel was observed at a lower flow rate of 10 mL/min. We could see the rapid contraction of the polymer matrix when these gels were heated to over the gel collapse point. The gel collapsed, and as a result, it was followed by a more gradual release of MB molecules from the restrained gel, both physically and figuratively. The hydrogels took 3 h to release the absorbed MB molecules at all three temperatures, showing that the regulated and sustained release was a result of the time required for MB molecules to diffuse from the hydrogel to the flow of the concentric tube.

**Figure 9:**
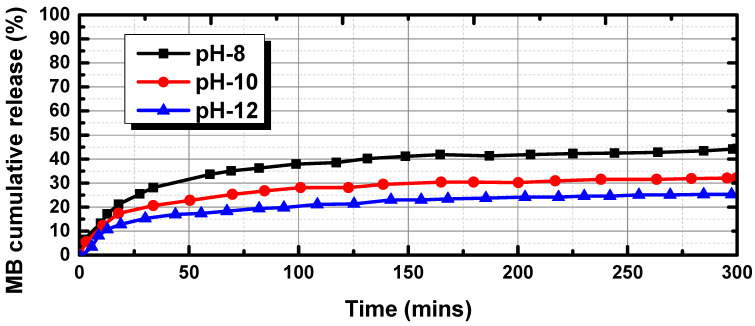
Comparison of the cumulative amount of MB released as a function of time at different pH values.

To investigate the impacts of pH-responsive behavior on regulated MB delivery, the MB release profiles were tested at 32 °C under varied pH values (pH 8, pH 10, and pH 12), as shown in Figure 9. The release rates considerably increased when the pH dropped from 12 to 8, showing that the pH of the medium had a major impact on the release of MD from the hydrogel. Only 19% of the MB was released at pH 12 after 50 min, and after that, the release rate remained nearly constant until about 26% of the MB was released after 300 min. Because of the partial protonation of the tertiary amine groups of DEA, which led to swelling, the release rates of MB accelerated at pH 8, with 40% of the MB being released after 100 min.

## 5. Summary and Conclusions

In this article, a compact liver mass and heat transfer device was tested on concentric double layer serpentine tubes by using the biomimicry method. The thermal performance of the liver-biomimicry polymer heat exchanger (PHE) consists of a hydrogel for regulated mass transfer, which is dictated by the determined fluid temperature flow inside the serpentine tubes. The designed PHE is produced by additive manufacturing technology using nylon 12. To execute the liver-based function within the device, temperature- and pH-regulated MB release was carried out in the new heat exchanger prototype. The experiments were carried out for 5 h. All calculations were made by using the hot fluid as the process fluid. The released MB was measured using UV/Vis spectroscopy and analyzed as a function of time, for different flow rates and fluid temperatures. The results of this study can be generalized as follows:

- The weight of the hydrogel was maximum at 10 mL/min and decreased by 25.29% to 10.12 g for the flow rate of 50 mL/min. On the other hand, the increase in flow rate to 50 mL/min delivered the maximum pressure drop of 7.21 kPa.
- The cumulative MB release at 30 °C increased to 47% at the lower flow rate of 10 mL/min, and the cumulative release at 40 °C climbed to 55%, which is 44.7% more than at 30 °C.
- The MB release rates considerably increased when the pH dropped from 12 to 8, showing that the lower pH had a major impact on the release of MD from the hydrogel. Only 19% of the MB was released at pH 12 after 50 min, and, after that, the release rate remained nearly constant until about 26% of the MB was released, after 300 min.
- The results show that hydrogels tested at higher fluid temperatures (40 °C) were quicker to de-swell and lost more water over time when compared to hydrogels tested at lower fluid temperatures (28 °C). At higher fluid temperatures, hydrogels lost approximately 80% of their water in just 20 min, compared to 50% at room temperature.
- From critical observations, it can be found that the impact of the weight gain of the hydrogel, due to swelling at an increased flow rate, is less than that of the increased pressure drop. At a lower flow rate, the hydrogel’s swelling over time is reflected in the increase in the weight of the hydrogel. We observed this behavior due to the pre-constructed walls that alter the swelling characteristics, due to which the hydrogel ultimately did not reach its final volume.

A compact mass-transfer device prototype based on liver function has been demonstrated in this work. Secondly, a technique for integrating the hydrogel into the device with a concentric fluid handling channel is shown. Additionally, MB release was regulated by a dynamic device design that can change the temperature demanded for by the hydrogels. Further, this design and functional parameters can be effectively optimized, in order to develop a potentially accelerated test setup for cancer drug testing that has vital biological functions, which could be used to deliver substantial contributions in the field of artificial organs.

## Figures and Tables

**Table 1:** Summary of the different hydrogels used for biomedical applications.

Author	Hydrogel	Compatibility/Application
Ji et al. [45]	Polyacrylamide by silver ion catalyzation	Biocompatible/tissue adhesion and Wound dressing
Saygili et al. [46]	Alginate-poly(acrylamide) hydrogel with TGF-β3 loaded nanoparticles	Biodegradability, biocompatible, and protein adsorption/cartilage repair
Zarrin et al. [47]	Polyacrylamide	Biodegradability/calvarial bone defect healing
Akkaya et al. [48]	Chitosan-poly(acrylamide-maleic acid)	Biocompatible/anticancer (MCF-7) drug doxorubicin targeted
Lv et al. [49]	Poly(acrylamide) with microsized β-chitin fiber	Biocompatible/arthritis treatment

**Table 2:** Design parameters of the liver heat and mass exchanger device.

S. No	Design Parameters	Dimensions (mm)
1	Length of the chamber (L_1_)	85
2	Breadth of the chamber (L_2_)	73
3	Chamber wall thickness (T_w_)	2
4	Outer diameter of the outer tube (Ot_od_)	10.50
5	Outer diameter of inner tube (Ot_id_)	8.50
6	Inner tube outer diameter (It_od_)	6.50
7	Inner tube inner diameter (It_id_)	5
8	Centerline distance between two tubes (d_1_)	11
9	Centerline distance between the first and second layer of two tubes (l_1_)	11
10	Height of the upper chamber (h_uc_)	23
11	Height of the lower chamber (h_lc_)	7
12	Diameter of the hot water fluid inlet (d_in_)	4
13	Total height of the chamber (h_c_)	38
14	Diameter of the bronze disc (d_bd_)	4
15	Diameter of the bronze disc (h_bd_)	5
16	Diameter of the orifice (d_o_)	1.50
17	Distance between two orifices (D_o_)	10
18	Outer tube bend radius (Ot_br_)	10.75
19	Inner tube bend radius (It_br_)	2.25

## Data Availability

The datasets used and/or analyzed during the current study are available from the corresponding author upon reasonable request.
